# Dataset of computed N-value and factual N-value traced for Soil Subsurface Profiling

**DOI:** 10.1016/j.dib.2020.105868

**Published:** 2020-06-16

**Authors:** Hisyam Jusoh, Teh Sabariah binti Abd Manan, Salmia Beddu, Syed Baharom Syed Osman, Muhammad Noor Hazwan Jusoh, Wan Hanna Melini Wan Mohtar, Taimur Khan, Nur Liyana Mohd Kamal, Abdulnoor A. Ghanim, Marzuki Ismail, Mohd Tajuddin Abdullah

**Affiliations:** a Geo TriTech, No. 17, Persiaran Perdana 15A, Pinji Perdana, 31500, Lahat, Perak, MALAYSIA; b Institute of Tropical Biodiversity and Sustainable Development, Universiti Malaysia Terengganu, 21300 Kuala Terengganu, Terengganu, MALAYSIA; c Department of Civil Engineering, Universiti Tenaga Nasional, Jalan Ikram-Uniten, 43000 Kajang, Selangor Darul Ehsan, MALAYSIA; d Department of Civil & Construction Engineering, Faculty of Engineering & Science, Curtin University, CDT 250, Miri, 98009, Sarawak, MALAYSIA; e Civil Engineering Department, Faculty of Engineering and Built Environment, Universiti Kebangsaan Malaysia, 43600 Bangi, Selangor, MALAYSIA; f Department of Civil Engineering, Faculty of Engineering, Najran University, P.O Box 1988, King Abdulaziz Road, Najran, SAUDI ARABIA

**Keywords:** Geotechnical, Investigation, Multi-channel analysis surface wave (MASW), Seismic, Subsurface, Statistic, Standard penetration test (SPT)

## Abstract

Soil requires load bearing impact assessment for stability. Therefore, this study aims to utilize the multi-channel analysis surface wave (MASW) for soil subsurface investigation and profiling around Peninsular Malaysia. The standard penetration test (SPT) was conducted for comparison between factual *N-value* and computed *N-value* from shear wave velocity (*V_s_*) obtained from MASW using the Imai and Tonouchi equation. The correlation coefficient (*R*) and coefficient of determination, (*R^2^*), showed strong relationship between factual *N-value* and computed *N-value*. The model of *V_s_* and factual *N-value* data distribution is non-normal but the analyzed relationship shows a significant level of *p-value* < 0.05. The *R^2^* for each location of *V_s_*-*N-value* relationship are ranging from 0.5 to 0.9.

Specifications table

**Table:** 

Subject	Civil Engineering and Engineering Geology
Specific subject area	Geotechnical Engineering
Type of data	Graph, Table
How data were acquired	Instruments Multichannel Analysis Surface Wave (MASW) survey with 24 channels system, 4.5 hz geophones, 1000 µs sampling rate and 2048 ms data length Standard Penetration Test (SPT)
Data format	Raw data (.sg2), filtered, analysed, computed
Parameters for data collection	Rayleigh wave (ground roll) Shear wave velocity (m/s) Blow counts (N-value)
Description of data collection	The multi-channel analysis surface wave (MASW) survey was conducted in the same location where the boreholes were drilled, and the bore-logs were recorded from Standard Penetration Test (SPT). The MASW survey data were processed and analysed for shear wave velocity (*Vs*) to compute *N-value.*
Data source location	Company: Geo TriTech, No. 17, Persiaran Perdana 15A, Pinji Perdana, 31500, Lahat, Perak Darul Ridzuan Country: Peninsular Malaysia Latitude and longitude (and GPS coordinates) for collected data: (Shah Alam - 3°05’16.5”N 101°33’09.9”E); (Bandar Puteri Jaya - 5°36’36.1”N 100°33’05.6”E); (Universiti Teknologi Petronas - 4°22’52.9”N 100°57’54.2”E); (Cyberjaya - 2°55’02.3”N 101°37’45.7”E); (Universiti Islam Antarabangsa Malaysia - 3°15′2.06"N 101°43′51.93"E); (Bangi - 2°58′8.25"N 101°46′58.66"E); (Meru - 3° 6′31.93"N 101°26′36.24"E); (Pudu Ulu - 3°07’21.9”N 101°44’08.6”E)
Data accessibility	With the article

## Value of the data

• The dataset present alternative method of load bearing impact assessment for soil stability that is useful for various applications requiring soil subsurface investigation and profiling
• The data will benefit civil engineers particularly geotechnical engineers providing data sourcing and tabulation during field works at large scale.
• The data will give added value for public safety in terms of land development based on further soil profiling and reliability studies.

## 1 Data Description

**Fig. 1:**
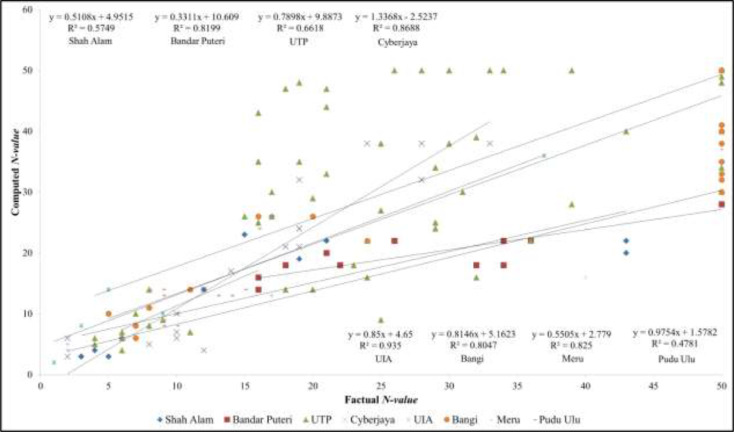
Computed *N-value* traced based on the location of the borehole in the survey line and comparison with factual *N-value*

The multi-channel analysis surface wave (MASW) was utilized to obtain shear wave velocity, *Vs* and computed *N-value* for subsurface profiling through Peninsular Malaysia. A statistical analysis was conducted to compare the factual and computed *N-value*. Statistical analysis indicates the correlation is significant at the level 0.01 (*p-value* < 0.05). Reliability and applicability of the proposed approach for computed *N-value* from *Vs* using Imai and Tonouchi [1] equation was verified from recorded bore-log of in-situ Standard Penetration Test (SPT) by comparing with uncorrected *N-value*. This verification is important since the study is trying to implement MASW as supporting to conventional method in order to reduce the limitations happen during soil/subsurface investigation [2]. To do so, there are eight locations involved for verification purpose. The factual *N-value* combined with depth and number of boreholes was compared with computed *N-value* from each location. The correlation and regression was attempted and equation was generated in this study. In general, it can be said that the correlations produced from factual-computed *N-value* were found moderate to strong coefficient of determination, *R^2^* ranging from 0.4 to 0.9 as depicted in Fig. 1. Both shear wave velocity and *N-value* from each location were plotted also for correlation and regression. Based on the data points, the following power-law expressions are obtained and broadly divided as demonstrated by previous researchers [3]. As can be seen in Table 1, the following equations were obtained between *V_s_* and *N-value* with the coefficient of determination, R^2^ are ranging from 0.5 to 0.9 indicating a moderate to strong relationship between these two variables.

**Table 1:** Summary of correlation and regression from each location in Peninsular Malaysia between shear wave velocity, *Vs* and factual *N-value*

Site Location	Regression (equation)	Coefficient of Determination, R^2^
Shah Alam	*Vs* = 41N^0.513^	0.7665
Bandar Puteri Jaya	*Vs* = 158.98N^0.1367^	0.6033
Universiti Teknologi Petronas	*Vs* = 92.352N^0.3425^	0.7432
Cyberjaya	*Vs* = 102.2N^0.3019^	0.7568
Universiti Islam Antarabangsa Malaysia	*Vs* = 145.95N^0.1757^	0.8715
Bangi	*Vs* = 119.65N^0.2588^	0.8405
Meru	*Vs* = 121.24N^0.1962^	0.9593
Pudu Ulu	*Vs* = 66.056N^0.4652^	0.5102

## 2 Experimental Design, Materials, and Methods

The seismic imaging survey was conducted using OYO Mc-Seis of 2D multi-channel analysis surface wave method (MASW). The survey was applied in the same location where the boreholes were drilled, and the bore-logs were recorded for uncorrected *N-value*. The *N-value*s were recorded from standard penetration test based on code of practice of BS 1377: Part 9. The shear wave velocity obtained from MASW survey provides a reliable and consistency value of material below the surface that can be used to compute *N-value* and hence giving a general view of subsurface profile [4].

There are three steps involve to obtain shear wave velocity profile from surface wave method; ground roll (Rayleigh wave) acquisition, construction of dispersion curve (phase velocity against frequency) and inversion or back calculation of the *Vs* profile from the calculated dispersion curve [5]. The MASW method also provides a two-dimensional profile (*2D*) of the shallow subsurface that was constructed through combination of one-dimensional (*1D*) shear wave velocity profiles .

In the analysis process, the shear wave velocity, Vs values were computed into *N-value*. The computed *N-value* was extracted from a well-established correlation of *Vs* against *N-value* incorporated in the software program which based on the studies conducted by Imai and Tonouchi, 1982 [6].

## Declaration of Competing Interest

The authors declare that they have no known competing financial interests or personal relationships which have, or could be perceived to have, influenced the work reported in this article.

## Appendix Supplementary materials

Image, application 1
